# Multimodal electrolyte architecting for static aqueous zinc-halogen batteries

**DOI:** 10.1093/nsr/nwaf029

**Published:** 2025-01-23

**Authors:** Tao Xiao, Jin-Lin Yang, Dongliang Chao, Hong Jin Fan

**Affiliations:** School of Physical and Mathematical Sciences, Nanyang Technological University, Singapore 637371, Singapore; School of Physical and Mathematical Sciences, Nanyang Technological University, Singapore 637371, Singapore; Laboratory of Advanced Materials, Shanghai Key Laboratory of Molecular Catalysis and Innovative Materials, State Key Laboratory of Molecular Engineering of Polymers, College of Chemistry and Materials, Fudan University, Shanghai 200433, China; School of Physical and Mathematical Sciences, Nanyang Technological University, Singapore 637371, Singapore

**Keywords:** Zn metal, aqueous batteries, synchronous electrolytes, halogen chemistry

## Abstract

Rechargeable static aqueous zinc–halogen batteries (AZHBs) thrive in energy-storage applications due to their suitable redox potential, abundant reserves and relatively high energy density. This non-flow battery relies on the collaboration of the reversible stripping/plating process of Zn metal and the halogen-participating zincation reactions. However, the corrosion of Zn metal and the shuttling of the halogen species result in serious capacity decay, posing challenges to their reversibility and lifespan. Moreover, the instability of high-valence halides hinders the implementation of multi-electron reactions in AZHBs. This review elaborates on the fundamentals, challenges and recent progress in AZHBs, highlighting the significance of the electrolyte design that is aimed at synchronous optimization for both the halogen cathode and the Zn anode in AZHBs. We discuss the design principles and protocols, along with concerns in the effective testing and evaluation of synchronous electrolytes. Possible approaches towards synchronous electrolytes are proposed—namely, biphasic electrolytes, gradient hydrogel electrolytes and ionic liquid electrolytes. This review may help to guide the research in achieving AZHBs with high energy density and longevity for practical applications.

## Teaser

This review looks at the challenges and solutions for designing advanced electrolytes to make long-lasting aqueous zinc-halogen batteries.

## INTRODUCTION

Rechargeable aqueous metal-ion batteries have garnered broad attention and are promising alternatives to lithium-ion batteries due to their inherent safety and environmental benignity. Alkali metals such as Li, Na and K generally cannot withstand the intrinsic electrochemical voltage window of aqueous solutions (1.23 V) despite their extremely low redox potentials [1]. Zn stands out as an ideal candidate for a metal anode due to its suitable redox potential (−0.76 V vs standard hydrogen electrode (SHE)), stable stripping/plating process in mild electrolytes, decent theoretical capacity (820 mAh g^−1^ and 5855 mAh cm^−3^) and earth abundance. To pair with the Zn anodes, conversion-type cathodes represented by halogens and their derivatives enable electron transfer through the switch between the elemental and ionic states. Consequently, the capacity that is contributed by this process is directly related to the change in the valence state of the halogens rather than the number of embedded charges in the intercalation scenario. Hence, halogen cathodes could provide higher capacity than conventional intercalation materials, especially considering the involvement of multi-electron reactions.

An aqueous solution is seemly simple. Unfortunately, Zn metals and halogens both suffer from poor reversibility in water-containing electrolytes. Zn inevitably encounters thermodynamically favored water-side reactions [2]. The hydrogen evolution reaction (HER), parasitic reactions and dendrite formation impose risks of battery short. For the halogens, thermodynamic instability and complex conversion result in intrinsic unsatisfactory reaction kinetics and reversibility [3,4]. The elemental halogens cannot exist stably in electrodes or electrolytes and the halogen intermediates that are generated in the conversion process will cause shuttle effects and self-discharge. Additionally, the high-valence halides that are derived from multi-electron reactions tend to induce low coulombic efficiency and fast capacity fading.

Strategies such as artificial protective layers [5,6], electrolyte additives [7,8], modified separators [9] and halogen-philic hosts [10] have been employed in aqueous zinc–halogen batteries (AZHBs) to alleviate the corrosion of the Zn anodes and stabilize the halogen cathodes. However, current reports generally prioritize modifications on one side of the electrode, rarely considering the synchronous enhancement for both sides. To tie all the knots, it is imperative to rethink how the aqueous electrolytes should be made ‘complicated’ without the sacrifice of cost and safety. In this short review, we summarize the current issues and optimization strategies of electrolytes pertaining to Zn–halogen systems, with an emphasis on the simultaneous electrolytes optimization for both the Zn side and the halide components (denoted as a synchronous electrolyte). Then, the principles and protocols of the synchronous electrolyte design are elaborated upon, followed by feasible approaches to realize for a synchronous electrolyte for AZHBs towards grid-scale energy storage.

## FUNDAMENTALS AND CRITICAL ISSUES IN THE AZHB

### Principles and reaction mechanism

**Figure 1.**
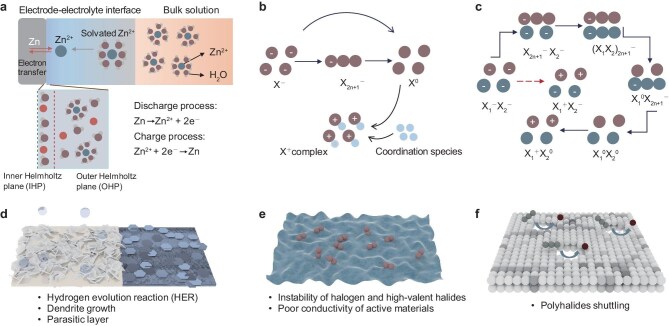
Fundamental redox chemistry and critical issues of aqueous Zn–halogen batteries. (a) Schematic of stripping-plating process of Zn anodes and interfacial chemistry. Illustration of (b) monohalogen conversion and (c) mixed halogen co-conversion mechanism. (d) Typical issues in Zn anode for AZHBs, including hydrogen evolution reaction (HER), parasitic reaction and dendrite growth. (e) Thermodynamics and reaction kinetics issues in the halogen cathode. (f) Shuttle effect of polyhalides within the electrolyte.

For the anode side of AZHBs, Zn^2+^ undergoes a cyclic deposition/dissolution process. During the charging process, which is driven by the electric field, solvated Zn^2+^ migrates from the bulk electrolyte towards the anode interface and then penetrates the electrical double layer (EDL) (Fig. 1a). Within the EDL, the solvated Zn²⁺ first undergoes the desolvation process, followed by adsorption, diffusion and charge transfer, and is ultimately reduced to Zn metal [11].

The halogen-related reactions on the cathode side are relatively complex and can be categorized into three modes based on the halogen type and electron transfer, which include single-electron mode and multi-electron mode involving only a single halogen, as well as co-conversion mode with multiple halogens involved.

In the single-electron conversion mode (Fig. 1b), cathode capacity is stored and released through reversible conversion of X^−^/X^0^. However, this process typically unfolds in multiple stages in real scenes due to slow reaction kinetics. Various intermediates, typically polyhalides, are gradually formed and converted with partial electron transfer. The generation of intermediate arises from (i) preferential reaction paths during the charge and discharge processes under the premise of slow kinetics (poor conductivities of both the charge and discharge final products) and (ii) disproportioned reactions between the halogen in the elemental and ionic states under static conditions. The subsequent reactions of the polyhalides depend on specific chemical environments.

In the multi-electron conversion mode, high-valent halogen compounds offer opportunities for multi-electron reactions, boosting cathode capacity by transitioning elemental halogen into high-valent halides. Unfortunately, these reactions tend to be irreversible due to the hydrolysis of high-valent halides in the aqueous electrolyte. Consequently, specific electronegative anions or groups are needed to coordinate with the positive halide ions [12].

The co-conversion mode that involves multiple halogens is more complex and intriguing (Fig. 1c), and is where various interhalogen compounds are formed. Halogen species can coordinate with each other and form stable complexes in this mode, thus avoiding the formation of irreversible products and providing multiple redox couples. In this mode, conversion proceeds stepwise, with the sequential observation of different valence states of each halogen, while maintaining the sequence of redox couples and theoretical capacity limits.

### Critical issues encountered in the AZHB

The Zn anode primarily suffers from HER, parasitic reactions and dendritic growth (Fig. 1d). Zinc metal is thermodynamically unstable in aqueous solutions. In neutral and weakly acidic electrolytes, it undergoes slow self-corrosion and hydrogen gas evolution. Localized pH fluctuation induced by HER increases the OH^−^ concentration, triggering parasitic reactions and inert byproduct accumulation. These insulating and disordered byproducts continuously consume zinc metal and electrolyte, adversely affecting the interface kinetics of the stripping/plating process. This spontaneous corrosion worsens the surface irregularities on the zinc metal, leading to uneven interface electric fields and randomly distributed energetic deposition sites. During deposition, protrusions with highly localized electric fields tend to attract more Zn^2+^, facilitating the dendritic growth that can cause a battery short. Additionally, the petal-like and porous regions will accelerate the HER and side reactions due to the increased surface area.

For the halogen cathodes, the intrinsic physicochemical nature hinders stable storage in the host materials and poses challenges to other battery components. For example, the volatility and corrosion of the battery components of chlorine gas raise concerns for safety and stability [13]. In addition, most halide compounds suffer from poor conductivity and the highly conductive host material is indispensable. As a result, halogen cathodes usually show low mass loading (typically <10 mg cm^−2^), leading to relatively low energy density and power density compared with intercalation-type cathodes (Fig. 1e). Further, the halogen-conversion process exhibits sluggish redox kinetics, along with uncontrolled mass and electron transfer at the interface, which inevitably result in the formation, dissolution and shuttling of reaction intermediates. Most intermediates exist in polyhalides, which are prone to react with zinc metal driven by chemical potential, leading to anode corrosion and self-discharge (Fig. 1f). Accompanying the deterioration of coulombic efficiency and reversibility, the electrons tend to transfer to the electrolyte, thereby reducing electrochemical stability. Simultaneously, certain electrochemically inert intermediates fail to undergo the subsequent conversion process, instead accumulating at the electrode–electrolyte interface and impeding charge- and mass-transfer processes [14]. Furthermore, multi-electron reactions that involve halogens require a wider electrochemical voltage window of the electrolyte. The high redox potential easily overlaps with the oxygen evolution reaction of the water. Additionally, due to the susceptibility of high-valent halides to hydrolysis and decomposition, the pH and composition of the electrolyte must be carefully controlled [15].

## RECENT ADVANCES AND OPTIMIZATION MECHANISM IN ELECTROLYTE DESIGN

**Figure 2.**
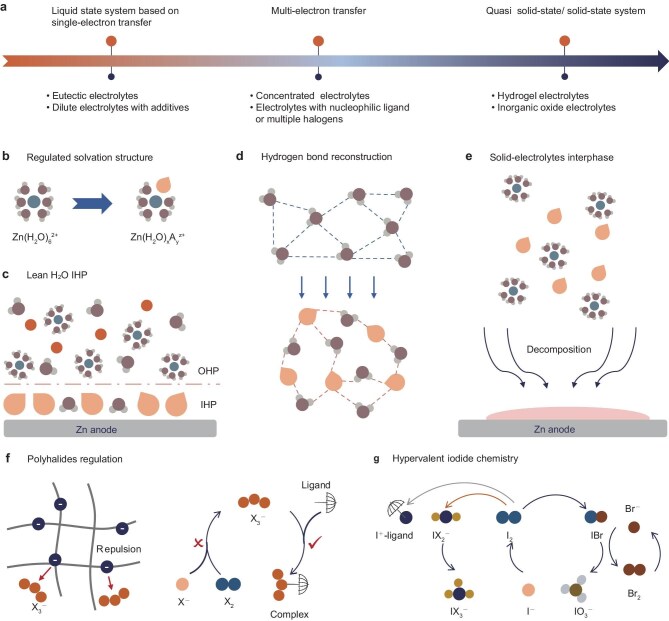
Recent advances and optimization strategies in the design of electrolytes (mostly non-synchronous). (a) Progress in the design of electrolytes for AZHBs. Role synopsis of reported electrolyte strategies in the Zn anode: (b) regulation of Zn^2+^ solvation structure, (c) adsorption optimization for lean H_2_O interface, (d) H-bond reconstruction and (e) SEI formation due to electrolyte decomposition. Optimization strategies for halogen cathodes: (f) suppressing polyhalides migration by electrostatic repulsion and capturing of polyhalides by ligands, (g) halide or strong nucleophilic ligand-mediated hypervalent iodine conversion.

To alleviate the corrosion of the Zn metal and improve the reversibility of the halogen cathode conversion, extensive strategies on electrolytes have been attempted (Table 1). They can be categorized into three main types (Fig. 2a): (i) liquid electrolytes involving a single halogen; this type is commonly seen in the design of electrolyte additives or eutectic electrolytes; (ii) liquid electrolytes with the involvement of multiple halogens; this approach relies on specific coordination species and highly concentrated electrolyte systems; (iii) solid or quasi-solid electrolytes in which only one halogen is involved in the reaction.

**Table 1.** Representative work of electrolyte optimization for aqueous Zn–halogen batteries.

Composition	Type	Anode stability	Cathode stability	Highlights	Ref.
BE + TAH	1	10 mAh cm^−2^; 1000 h	2 A g^−1^; 5000 cycles	I^−^/I^0^/I^+^ reaction in dilute electrolytes	[16]
ZnCl_2_ + LiCl + ACN	2	1 mAh cm^−2^; 1000 h	2 A g^−1^; 6000 cycles	I^−^/I^0^/I^+^ conversion in concentrated ZnCl_2_ system	[12]
30 m ZnCl_2_	2	1 mAh cm^−2^; 200 h	2 A g^−1^; 6000 cycles	Three-electron reaction based on interhalogen compounds ICl_3_^−^	[17]
BE + H_2_SO_4_ + KBr	2	2 mAh cm^−2^; 800 h	2 A g^−1^; 150 cycles	Twelve-electron conversion with multiple halogens	[18]
ZnCl_2_ + ZnSO_4_ + HPYBr	2	2 mAh cm^−2^; 600 h	3 C; 1000 cycles	Br^−^/Br^0^/Br^+^ conversion enabled by ILs	[19]
DMS–Zn(ClO_4_)_2_–NA	2	0.5 mAh cm^−2^; 2000 h	2 A g^−1^; 2000 cycles	Hypervalent iodine conversion in eutectic electrolytes	[20]
BE + Mullite	3	0.1 mAh cm^−2^; 140 h	0.5 A g^−1^; 3000 cycles	All-solid-state Zn–I_2_ battery	[21]
PAH–PCH hydrogel	3	1 mAh cm^−2^; 3000 h	8 C; 15 000 cycles	Hydrogels for synergistic enhancement in iodine batteries	[22]
Zn(OTf)_2_–N–ACE	1	0.5 mAh cm^−2^; 350 h	4 mA cm^−2^; 5000 cycles	Eutectic electrolytes for Zn–I_2_ batteries	[23]
BE + EMIMOAc	1	1 mAh cm^−2^; 3000 h	4 A g^−1^; 18 000 cycles	ILs for synergistically optimized Zn–I_2_ batteries	[24]
Zn(DFTFSI)_2_	1	4 mAh cm^−2^; 2500 h	2 A g^−1^; 1200 cycles	*In situ* SEI for Zn-anode protection and shuttle effect inhibition	[25]
BE + MPIBr	1	1 mAh cm^−2^; 2000 h	2 mA cm^−2^; 1000 cycles	ILs for simultaneous enhanced Br_2_ batteries	[26]
P(AMPS–Zn) hydrogel	3	1 mAh cm^−2^; 5000 h	2 C; 2000 cycles	Single-ion conductor for Zn–I_2_ batteries	[27]
BE + TEA	1	1 mAh cm^−2^; 1800 h	10 A g^−1^; 35 000 cycles	Janus interface for Zn–I_2_ batteries	[28]
BE + PEG	1	1 mAh cm^−2^; 500 h	0.4 A g^−1^; 1500 cycles	I^−^/I^0^/I^+^ reaction over a wide temperature range	[29]
ZnCl_2_	2	NA	1 C; 400 cycles	I^+^/I^3+^ conversion	[30]
LiCl	1	NA	0.5 A g^−1^; 100 cycles	Cl^0^/Cl^−^ redox couple under low temperature	[31]
LiCl + LiBr	1	NA	0.2 C; 400 cycles	Intercalation and co-conversion chemistry of Br–Cl	[32]

BE refers to the basic electrolyte that is composed of zinc sulfate and water.

Based on Table 1, we then elaborate on the optimization strategies for both the Zn anode and the halogen cathode sides, respectively.

### Regulation of solvation structure, hydrogen bond and interface chemistry for Zn anodes

For Zn anodes, the core objective is to reduce the activity and the number of interface H_2_O molecules. The four mainstream strategies can be summarized as: (i) Zn^2+^ solvation structure regulation, (ii) hydrogen-bond reconstruction, (iii) interfacial adsorption and inner Helmholtz plane (IHP) regulation and (iv) solid–electrolyte interphase (SEI) construction.

- Solvation structure regulation. In dilute aqueous electrolytes, the Zn^2+^ primarily exist as solvent-separated ion pairs within the electrolyte, meaning that the Zn^2+^ are fully surrounded by H_2_O molecules, excluding anions into the loose secondary solvation shell. Such a water-rich primary solvation shell will foster the generation of hydrogen gas. Various electron-donating organic molecules or functional groups, which exist as additives [33], hydrogels [34] or deep eutectic solvents [35], have been incorporated into aqueous electrolytes to attenuate the inherent Zn^2+^–H_2_O molecule interactions, thus reducing the coordination number of H_2_O molecules in the solvation structure and enhancing the overpotential for HER (Fig. 2b). The key to this competitive mechanism lies in the strong affinity between the specific additives and the functional groups with Zn^2+^, particularly the Lewis basicity and the ability to provide lone electron pairs. Consequently, the functional units are often rich in N, O, S, P or halogen species.
- Interfacial absorption and IHP modulation. The interfacial electrochemistry of the Zn anode also highly impacts the stripping/plating process. The anode–electrolyte interface is dominated by the EDL, which is composed of the IHP and the outer Helmholtz plane (OHP). The IHP water dipole is expected to be compressed or even excluded to the OHP to circumscribe the side reactions under static conditions and bias (Fig. 2c). Such a lean-H_2_O IHP can be achieved by increasing the concentration of the zinc salts or introducing specific additives. In concentrated electrolytes, more anions can overcome electrostatic repulsion and aggregate on the anode surface, thereby squeezing water molecules out of the IHP [36]. Alternatively, additives with strong adsorption to Zn metal can also create a lean-H_2_O interface [37]. The specific adsorbents in this strategy are independent of the charge species, but they should render a higher zincophilicity than water molecules and be hard to be desolvated. Common metal cations or Lewis bases that readily coordinate with Zn^2+^ are not preferred; instead, bulky cations with dispersed charges or weakly solvated hydrophobic anions are more suitable. In addition to inhibiting the side reactions that are derived from the water dipoles in the IHP, this additional adsorption layer is also favorable to guide the uniformized Zn deposition by adsorbing on the protrusions during Zn deposition [38]. Moreover, this adsorption feature may be preferred for specific facets, resulting in prioritized growth with a specific orientation [39].
- Hydrogen-bond reconstruction (Fig. 2d). Most H_2_O molecules in the electrolyte form a hydrogen-bonded tetrahedral network. H^+^ and OH^−^ can rapidly transport through the ordered long-range H-bond network based on the Grotthuss diffusion theory, facilitating the HER and accelerating the corrosion of the Zn anode [40]. Hence, various additives with hydrogen-bond donors or acceptors are introduced [41]. These additives block proton conduction based on the tetrahedral H-bond network and effectively suppress subsequent proton adsorption on the Zn-anode surface. Another H-bond regulation strategy involves reducing the content and activity of the free water in the bulk electrolyte. Electrolyte systems such as concentrated electrolytes [42], water-in-ionic liquids [43] and hydrogels [44] serve to immobilize free H_2_O molecules, which can further broaden the electrochemical window but sacrifice ionic conductivity.
- SEI construction. The construction of an SEI aims to prevent direct contact between the electrolyte and the Zn metal, thereby eliminating HER and side reactions (Fig. 2e). The SEI derived from the electrolyte can be spontaneously formed at the interface. The hydrolysis products of the additives or Zn salts such as fluoroethylene carbonate, KPF_6_ and Zn(BF_4_)_2_ could spontaneously react with the Zn metal and form the hybrid SEI [45–47]. However, due to the high redox potential of Zn metal, the practical SEI usually originates from the decomposition of electrolyte components during the electrochemical process. This decomposition requires a lower lowest unoccupied molecular orbital (LUMO) energy level of Zn^2+^-anions/additive complexes to ensure the preferential reduction of additives or anions compared with the water [48]. Thus, this strategy typically relies on highly concentrated electrolytes or additives/anions that can participate in the first saolvation shell of Zn^2+^, have lower reduction potentials and can suppress the decomposition of the water molecules. An effective SEI is usually composed of an inorganic inner layer that is derived from salt decomposition and an organic or organic/inorganic composite outer layer from a solvent/additive reduction. The inorganic components [e.g. ZnF_2_, ZnS, Zn_3_(PO_4_)_2_] are mainly responsible for the rapid conduction of Zn^2+^ [49,50], while the organic components play a pivotal role in effectively homogenizing the ion flux towards uniform Zn deposition [51]. It should be noted that the true function of SEI, especially the Zn conduction within the SEI layer in an aqueous system, is still under debate.

### Polyhalide confinement and multi-electron reaction regulation of halogen cathodes

For reported halogen cathodes, the reaction mechanisms primarily involve single-electron and multi-electron processes. In the case of AZHBs that are based on single-electron reactions, the main challenge lies in the generation and dissolution of polyhalide intermediates. Therefore, the electrolyte optimization strategy focuses on limiting the polyhalide transport and generation (Fig. 2f). Given the high water solubility of polyhalides, relatively lean-water electrolyte systems such as water-in-salt electrolytes [52], deep eutectic electrolytes [53] and hydrogels [54] are employed to reduce the diffusion coefficient of the polyhalides towards the Zn anode. Additionally, charged functional groups can hinder the migration of polyhalides through repulsion interaction, as observed in hydrogel systems that are rich in COO^−^ and SO_3_^−^ [55,56]. Besides limiting polyhalide shuttling, another effective strategy is to modulate the reaction pathways and kinetics to inhibit polyhalide generation. For instance, iodide exhibits lower solubility and reduced hydration states in deep eutectic electrolytes [57] and concentrated electrolytes [58], leading to unfavorable thermodynamic conversion of polyiodides. In addition, specific complexing agents can effectively capture polyhalides and form hydrophobic intermediates, thus eliminating the possibility of polyhalides shuttling [19,24].

For AZHBs that rely on multi-electron reactions, we need activate and stabilize the highly hydrolysed high-valent halide ions. The high-valent conversion of iodide is a representative case for discussion. The introduction of additional halogen sources or strong nucleophilic species has proven effective in forming stable complexes with halogen cations (Fig. 2g), thereby preserving their electrochemical activity and preventing hydrolysis [20,59,60]. Additionally, increasing the upper potential limit during charging can enable the conversion of Cl^−^/Cl^0^ or Br^−^/Br^0^. In this process, interhalogen compounds formed by I^+^ and heterohalogen ions can also effectively stabilize the generated Br_2_ and Cl_2_. Furthermore, the highly reversible I^−^/IO_3_^−^ can be activated under acidic conditions with the aid of heterohalogen ions [61]. The Br^−^/Br^0^ redox couple can largely reduce the kinetic barriers of the iodate during charge and discharge. However, it should be noted that the above strategies usually rely on a water-poor electrolyte, as the formed complexes are highly water-sensitive due to the nucleophilic attack by the OH moieties of water molecules [62,63].

## SYNCHRONOUS ELECTROLYTES FOR ZINC–HALOGEN BATTERIES

**Figure 3.**
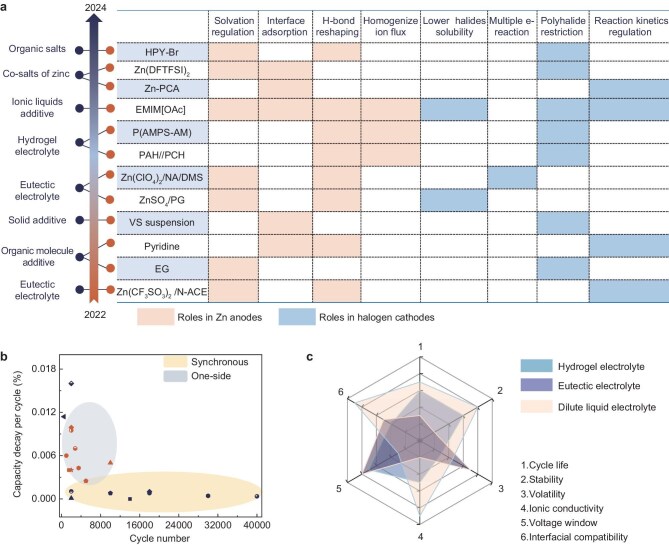
Overview of recent synchronous electrolytes for aqueous Zn–halogen batteries. (a) Functions of reported electrolytes in Zn anodes and halogen cathodes. (b) Full cell performance (capacity decay rate vs cycle number) of AZHBs with synchronous enhancement [20,24,54,55,64,65,67–70] and one-side optimization [71–73,157–162]. (c) Comparison between hydrogels, dilute electrolytes and eutectic electrolytes in terms of the critical properties of AZHBs.

For the current AZHBs system, most published papers are about designing electrolytes to mitigate self-corrosion on the Zn-metal side. On the halogen cathodes, the mainstream strategy involves designing highly conductive and halogen-philic hosts to confine active materials within the electrodes. Overall, previous efforts have been put into improving the reversibility and stability on a single electrode side, neglecting the simultaneous enhancement of both electrodes. Several reports have emerged recently to address the synchronous optimization of AZHBs by electrolyte modulation and these electrolyte systems are referred to as synchronous electrolytes. The strategies and mechanisms are summarized below (Fig. 3a). These strategies can be classified into three categories based on the type of electrolyte: electrolyte additives [19,24,25,64–67], eutectic electrolytes [20,57,68] and hydrogels [69,70].

Regarding electrolyte additives, they primarily serve the following purposes: (i) strong affinity with Zn^2+^, facilitating solid coordination with Zn^2+^ and reducing the water molecules in the first solvation shell; (ii) rich zincophilic functional groups, promoting preferential absorption towards the Zn metal; (iii) the presence of hydrogen-bond donors or acceptors, reshaping the hydrogen-bond network and reducing the number of free water molecules; and (iv) capability of complexing with halogen species to inhibit the formation or migration of polyhalides.

For eutectic electrolytes and hydrogels, the intrinsic low free water content endows them with the ability to inhibit the Zn-anode corrosion and the generation and shuttling of the polyhalides. Beyond the basic synergistic gain, the performance of the AZHBs can be further enhanced through the design of functional groups of the hydrogel and composition adjustment of the eutectic electrolyte. A typical example is the hetero-structured hydrogel reported by Yang *et al.* [70]. The hydrogel with abundant zincophilic SO_3_^−^ groups on the anode side can serve as an ion sieve to effectively homogenize the Zn^2+^ flux and guide uniform deposition, while the polycationic hydrogel on the cathode side can trap polyiodides and accelerate the conversion kinetics with the aid of the conductive interface. In eutectic electrolyte systems, H_2_O molecules almost all participate in forming a eutectic structure with a hydrogen-bond donor or hydrogen-bond acceptor rather than the tetrahedrons that comprise bound H_2_O. These properties broaden the electrochemical window, alleviating the corrosion of the Zn metal and enabling multi-electron conversion of the halogens with specific component optimization. For instance, Li *et al.* demonstrated a DMS–Zn(ClO_4_)_2_–6H_2_O–NA deep eutectic electrolyte; the I^+^ can be anchored by the nucleophilic niacinamide (NA) ligand, thus correspondingly achieving reversible I^−^/I^0^/I^+^ conversion [20]. In addition, thanks to the solvation structure of [Zn(ClO_4_) (DMS)_x_(NA)_y_(H_2_O)_z_], the Zn anode achieved an enhanced average coulombic efficiency and more homogeneous deposition.

Next, the performance of full cells of AZHBs based on synchronous electrolytes and unilateral optimization are summarized and compared (Fig. 3b). For studies focusing on unilateral optimization, the main approaches involve the modification and design of hosts that can contain the functions described below. Conductive hosts require a hierarchical pore structure with large surface areas to improve the loading capacity and enhance physical adsorption [71]. Additionally, due to polar–polar chemical interactions, hosts with specific surface functional groups exhibit a strong chemical affinity for elemental halogens and polar intermediates [72]. Beyond physical confinement and chemical adsorption, catalytic sites, such as those represented by transition metal atoms or clusters, can lower conversion barriers and accelerate redox kinetics [73]. A few reports have employed quasi-solid electrolytes with iodine-philic to limit the migration of polyiodides, effectively confining the polyiodides but at the expense of poor ionic conductivity [74]. Although these two strategies have achieved some degree of optimization for the duration of AZHBs, these systems lack any improvement for the anode corrosion. Consequently, the comparison shows that full cells using synchronous electrolytes exhibit superior cycle life and slower capacity decay compared with the one-side optimization. This result further underscores the importance and significance of synchronous optimization in AZHBs.

The selection of synchronous electrolyte systems currently focuses on three types: dilute liquid electrolytes, hydrogel electrolytes and electrolytes that are based on deep eutectic solvents (DES). The analysis and comparison of key parameters, such as ionic conductivity, stability, volatility, interfacial compatibility, electrochemical window and cycle life, are conducted in these three systems of AZHBs (Fig. 3c). Although DES-based electrolytes have the advantage of a wide electrochemical window, they exhibit different levels of inadequacies in other parameters. The water molecules in DES predominantly exist in the eutectic structure, resulting in an expanded electrochemical range with a sacrifice in ionic conductivity. The ionic conductivity of DES is generally <10 mS cm^−1^, which is considerably lower than that of conventional hydrogel and dilute electrolytes [75]. Furthermore, DES-based electrolytes generally exhibit a reduced Zn^2+^ transference number because of the primary contribution of non-zinc ion components to conductivity. Another important concern revolves around the choice of the zinc salt-in DES. Despite the enhancement of the coulombic efficiency of the Zn stripping and plating process that is endowed by eutectic electrolytes [76,77], their low thermal stability, volatility and corrosion to battery components limits their further application. Furthermore, the DES-based electrolyte is profoundly susceptible and slight changes in moisture or temperature can cause degradation and fluctuation of its electrochemical characteristics. Consequently, hydrogels and dilute electrolytes are ideal for promoting the design of synchronous electrolytes due to their acceptable conductivity, excellent chemical and thermal stability and rich design versatility.

## DESIGN PRINCIPLES AND EVALUATION OF SYNCHRONOUS ELECTROLYTES

In the previous section, we summarized the mechanisms and strategies associated with synchronous electrolytes for AZHBs. To further promote the practical application of Zn–halogen batteries, it is imperative to propose guidelines for the synchronous design of electrolytes for the next stage. The primary issue is how to rationally and systematically select, design and evaluate the effectiveness of electrolytes. In this section, we first raise concerns about the future testing and evaluation of AZHBs and then propose design principles and protocols for synchronous electrolytes.

### Concerns about testing and evaluation of Zn–halogen batteries

Although countless efforts have been made to improve the reversibility of Zn anodes, evaluating the effectiveness of electrolyte modifications is often neglected. Regarding the current reports, the evaluation of Zn-anode corrosion behavior typically includes electrochemical tests, half-cell tests and symmetric cell tests. For electrochemical tests, parameters such as the exchange current density, Zn^2+^ transference number and activation energy are employed as typical descriptors to explain the evolution of Zn-metal corrosion and Zn^2+^-migration behavior. However, it should be noted that the corrosion of Zn metal in aqueous electrolytes is thermodynamically spontaneous and continuous, making the influence of resting time on these descriptors inevitable. For example, Li *et al.* clarified the correlation between the resting time and the evolution of the byproduct ZHS, finding that the Zn anode displays the optimal lifespan at a specific resting time [78]. Likewise, the Zn^2+^ transference number also correlates with the resting time [79]. The lack of description in this parameter makes the performance in most reports incomparable and raises concerns about reliability. Therefore, specification of the resting duration of tests or use of the zinc-free test architecture in future evaluations is recommended. Specifically, it is recommended to rest the assembled battery to reach a charge-transfer resistance *R*_ct_ at its initial steady state. This approach not only eliminates potential changes caused by spontaneous corrosion during electrochemical testing, but also prevents the accumulation of inert products. Furthermore, specialized tests should employ zinc-free testing architectures, such as the linear sweep voltammetry tests and Zn^2+^ transference number measurement [80], as it does not require the use of zinc metal.

Another concern in evaluating Zn-anode performance is the choice of test parameters and methods, especially for Zn-deposition behavior, the long cycling tests of symmetric cells and the coulombic efficiency of half-cells. For the tests on Zn deposition and coulombic efficiency, low current density and low areal capacity (e.g. 1 mA cm² and 1 mAh cm²) should be prioritized over high current density and low areal capacity (e.g. 5 mA cm² and 1 mAh cm²). High current density induces textured deposition morphology [81] and masks the effect of HER [82], potentially leading to misjudgment of validity. High-current-density parameters are more suitable for evaluating dendrite suppression, particularly in Zn symmetric cells. Additionally, a high depth of discharge should be emphasized in symmetric cell tests, as it is vital for enhancing Zn-anode utilization and reducing the ratio of the negative electrode capacity to the positive electrode capacity (N/P ratio) of the full cell. The measurement of coulombic efficiency (CE) is also an issue worthy of attention. Conventionally, Zn plates are regarded as zinc sources and all Zn metal that is plated on the current collector is stripped to calculate the CE of each cycle. However, excess Zn masks the Zn loss during a single stripping/plating cycle, such as dead Zn or Zn shedding from the current collector. Hence, it is recommended to use a reservoir CE test to enhance accuracy [83]. This involves pre-depositing specific Zn on the current collector to serve as the ‘reservoir’ and the anode of the half-cell. Further, a resting process can be introduced into the half-cells between the discharging and charging processes to strictly evaluate the capacity loss caused by calendar aging in the Zn anode [84].

The evaluation of the optimization on the halogen side mainly involves the monitoring of the generation and evolution of polyhalides. Typically, spectral characterization during the charge and discharge process, the self-discharge test and the long cycle performance of full cells are used as indicators. However, the stripping/plating process on the Zn side may interfere with the above tests. For instance, the polyhalide shuttling may alleviate the dendrite growth to some extent, thus extending the cycle life and resulting in signal interference. Therefore, additional descriptors and tests regarding halide conversion are needed to evaluate the effectiveness of electrolyte modification on halogen-based cathodes. Given the similar conversion processes and mechanisms of halogens and sulfur, tests such as potentiostatic *I*–*t* test and cyclic voltammetry test of symmetric cells assembled with polysulfides [85] or descriptors including activation entropy and shuttle coefficient in lithium–sulfur batteries can also be considered for reference in AZHBs [86], which will help to clarify the reaction path and rate-controlling step. Furthermore, pouch cells with a low ratio of electrolyte/active material of halogen, high mass loading and high areal capacity should be developed to promote commercialization.

### Principles and protocols for synchronous electrolyte design

Previous reports lack clear and comprehensive guidelines for the optimization of AZHBs. Some descriptors have been proposed, such as the SEI capability of additives [87], Gutmann donor number [88], adsorption energy [89] and dielectric constant [90] through machine learning or experiments. Considering the distinct requirements of the zinc anode and halogen cathode, and the complexity of electrochemistry, these individual descriptors could be incomplete. Taking the Zn anode as an example, the optimized solvation structure requires components with strong affinity with Zn^2+^, while the lean-H_2_O IHP emphasizes the hydrophobic nature and strong adsorption to the Zn metal. Therefore, the identification of a single descriptor for the synchronous optimization of Zn–halogen batteries is a challenge.

In addition, given the high cost, high viscosity, stability and low ionic conductivity, there is limited room for the simultaneous design of eutectic electrolytes and concentrated electrolytes. Especially for halogen-conversion cathodes, the slow kinetics in eutectic electrolytes and concentrated electrolytes severely limit their long cycle and rate performance. Hence, hydrogels and dilute electrolyte systems are more suitable candidates as synchronous electrolytes, which offer acceptable ionic conductivity, high solubility of zinc salts and flexible composition adjustment. Here, we will propose the qualitative principles and protocols of electrolyte design in terms of hydrogels and dilute liquid electrolytes.

In terms of electrolyte structure, traditional electrolytes typically consist of a single phase or a single layer. Without precise composition adjustments, they are unable to fulfill the specific and multifunctional demands that are required by synchronous electrolytes. Consequently, decoupling the anode and cathode electrolytes in the structure will provide another pathway toward high-performance AZHBs. The hydrogel system is well suited for investigating the necessary conditions for structural decoupling, thanks to its acceptable ionic conductivity, adaptable functional groups and diverse combinations. By employing property gradients in a hydrogel with a single layer or by combining multilayered hydrogels, problems related to the Zn anode and halogen cathode sides can be tackled effectively. This structural decoupling can also be realized through multiphasic liquid electrolytes, which consist of phase domains with distinct solvents. Such phase separation can occur through either thermodynamically immiscible liquids or passive phase separation in miscible systems [91].

The electrolyte component is the core of the synchronous electrolytes, especially for liquid electrolytes. In this context, an electrolyte component that consists of a single zinc salt or a single solvent is inadequate. Instead, the electrolyte must encompass distinct functional components that can autonomously engage in the optimization of the Zn-metal stripping/plating process and halogen-conversion process. Ideally, these functional units should be weakly associated and exhibit multiple roles in an AZHB, including solvation, mass transfer and redox chemistry.

**Figure 4.**
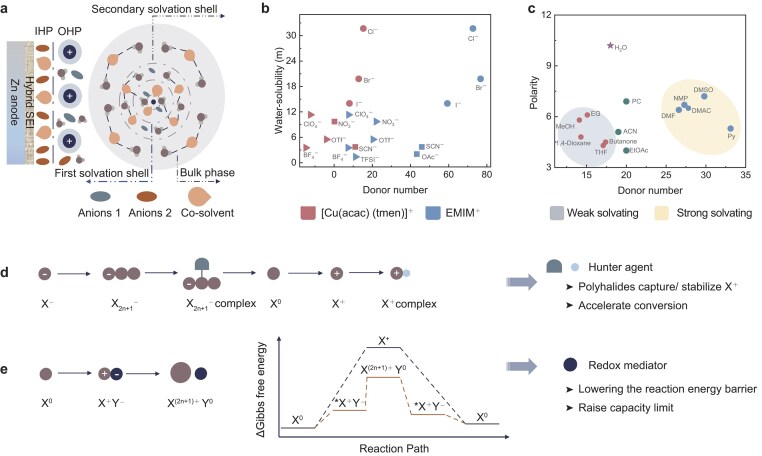
Principles and protocols of the design of synchronous electrolytes. (a) The ideal composition of the liquid electrolyte for AZHBs. (b) Summary of the Gutmann donor number (DN) for common anions in zinc salts and the corresponding aqueous solubility. Symbols with different colors correspond to the two ligand cations during the DN measurement. Scattered dots with various shapes depict different degrees of DN and solubility. (c) Overview of the Gutmann DN and polarities of various organic solvents. Optimized halogen reaction pathways utilizing (d) hunter agent and (e) redox mediator.

The ideal electrolytes should possess the following properties: water-poor Zn^2+^ solvation shell, the modulated hydrogen-bond network in the bulk phase and solvation structure, a lean-H_2_O IHP and SEI with both high ionic conductivity and hydrophobic properties (Fig. 4a). To fulfill these requirements, the electrolyte should basically consist of at least two zinc salts and a co-solvent. Anion 1 primarily mainly contributes to the creation of the Zn^2+^ solvation structure and SEI formation, reducing the number of water molecules in the solvation shell and isolating the contact with water, respectively. Anion 2 is expected to demonstrate a lack of affinity to Zn^2+^ and strong adsorption to zinc metal, facilitating its penetration into the IHP and the exclusion of water molecules at the interface. Thus, anion 1 should possess a high level of coordination capability towards Zn^2+^, which can be assessed by using the Gutmann donor number (DN) that indicates the electron-donating ability. Additionally, increased zinc salt concentration enhances Zn^2+^-anion coordination, making zinc salt solubility a crucial screening parameter. The DN of various common anions and the solubility of the corresponding zinc salts are summarized in Fig. 4b [92,93]. It can be found that halogen ions, OAc^−^ and SCN^−^ show high coordination ability and solubility, which make them ideal for participating in the solvation structure and building the SEI due to strong Zn^2+^-anion interaction. For anion 2, anions with low DN values such as BF_4_^−^, TFSI^−^, OTf^−^ and SO_4_^2−^ are suggested due to their poor solvation characteristics. Furthermore, anions with electron-withdrawing groups offer benefits in constructing the lean-H_2_O IHP. For example, hydrophobic and electron-withdrawing OTf^−^ displays a distinct EDL compared with hydrophilic SO_4_^2−^, which exhibits the distribution of more Zn^2+^ and fewer H_2_O molecules near the anode surface [94]. Although halogen ions possess notable solubility and coordination capability, they are better suited as trace additives rather than the primary constituent of the electrolyte due to the risk of corrosion to battery components. In addition, anions such as BF_4_^−^, NO_3_^−^ and SCN^−^ are also unsuitable as main compositions due to the lack of electrochemical stability and thermal stability. Overall, OAc^−^ is a suitable choice as anion 1 to constitute the main zinc salt component due to its strong coordination ability and high solubility. Its task is to generate an enhanced contact ions pair ratio in thesolvation structure and contribute to the formation of SEI. Anions such as OTf^−^ and TFSI^−^ with low DN are more appropriate as anion 2 to achieve a hydrophobic electrode–electrolyte interface.

For co-solvents, the DN and polarity of various solvents are summarized (Fig. 4c) [95]. Organic solvents with a high DN value and high polarity such as DMSO, DMF, NMP and DMAC are preferred as co-solvents for constructing the strong solvating electrolytes. These co-solvents further decrease water molecules in the solvation shell and are preferentially adsorbed on the IHP, thus reducing water on the Zn surface. Additionally, given the degradation and depletion of the inorganic phase-dominated SEI during cycling, an organic–inorganic hybrid SEI that is formed with co-solvents is necessary for effectively protecting the Zn metal [96]. Moreover, organic solvents with high DN and polarity can disrupt the bound water molecule network and hinder the process of HER [97]. In addition, recent reports indicate that some low-polarity organic molecules do not participate in the first solvation shell but can efficiently decrease the presence of water molecules in the Zn^2+^ solvation shell and intensify the Zn^2+^-anion coordination [98–100]. This weak solvating effect is crucial for creating solvation structures that incorporate weakly solvated anions such as SO_4_^2−^, OTf^−^ and TFSI^−^ at ​​low concentrations, which exhibit superiority in constructing an SEI with higher ionic conductivity and faster desolvation kinetics.

In addition to the mentioned anions and co-solvents, specific adsorbents can also be advantageous for further optimization. Molecules and anions with a strong affinity for zinc electrodes, such as sulfonic acid groups [101] and cysteine [102], can be incorporated into synchronous electrolytes. Specific adsorbed anions allow excess Zn^2+^ to enter the OHP, thus reducing concentration polarization and promoting interface kinetics [103]. Hydrophobic cations can be utilized to decrease water density in the EDL and facilitate the reduction of anions at the interface, which results in the formation of a protective SEI layer [104].

To resolve problems on the halogen cathode side, it is necessary to employ hunter agents and redox mediators. First, it is crucial to ensure that polyhalides that are generated during halogen conversion are securely anchored by additives (Fig. 4d). Organic ammonium cations and imidazolium cations exhibit a strong affinity to polyiodide ions and are prone to combining with them to form stable complexes. Notably, certain cations such as EMIM^+^, [N_2211_]^+^ and [N_2221_]^+^ can form water-insoluble ionic liquids with polyiodides [24,105], facilitating a fast conversion rate. In addition, halogen cations with high valence require stabilizers to prevent hydrolysis, which can be achieved by using organic ligands. The ligand should exhibit strong nucleophilicity to easily form charge-transfer complexes with electrophilic high-valent halogen species, thereby stabilizing the redox process. Previous studies have shown that nucleophilic compounds, such as quinoline, piperidine and pyridine, can stabilize I^+^ due to the existence of N atoms with lone pairs of electrons [106]. Consequently, strong nucleophilic halides (e.g. X^−^, where X = F, Cl, Br), cyanides and amines are potential candidates. Additionally, the ligand must exhibit considerable water solubility. While some organic ligands, such as Am_4_N^+^ and Hexy4N^+^, can form stable complexes with high-valent halogen species, poor solubility disqualifies them as suitable candidates [107]. Another factor that needs to be emphasized is the water content and pH of the electrolytes. The hydrolysis of high-valent halogen species remains a challenge, even in the presence of nucleophilic ligands. For example, the commonly used ligand Cl^−^ can stabilize the I^0^/I^+^ redox couple only in highly concentrated electrolytes [12]. To suppress the hydrolysis, electrolytes with low water content, high ligand concentration and strong acidity are necessary. Moreover, incomplete conversion of polyhalides during the X^−^/X^0^ redox process can neutralize the high-valent halogen species, thereby reducing the reversibility of the multi-electron reactions [108]. To address this, the organic ligand should anchor or inhibit the formation of halogen intermediates to a certain extent. Furthermore, the presence of redox mediators aims to reduce the redox potential of high-valent halogen species. Positive halogen ions encounter obstacles such as elevated oxidation potential, sluggish reaction kinetics and severe side reactions, rendering them problematic for utilization in AZHBs [61]. Therefore, it is worthwhile to investigate suitable redox mediators. The optimization mechanism is depicted in Fig. 4e. The redox mediator can coordinate with high-valent halogen ions and undergo electron transfer in the subsequent oxidation process, resulting in the formation of a stable complex. This distinct conversion path is capable of greatly reducing the reaction potential and energy barrier, allowing rapid kinetics and the high utilization of high-valent halogen compounds. The halide is a proven redox mediator that is used in the AZHBs system. During the charge and discharge process, multiple halides in the electrolyte and electrode might bind to each other, leading to the occurrence of reversible multi-electron reactions and enhanced CE. However, this strategy generally works well only in concentrated electrolytes, thus casting a shadow on the stability of the battery components. Thus, it is imperative, while challenging, to discover redox mediators that can function effectively in diluted electrolytes. In addition to halides, redox-active organic molecules can be explored as potential candidates for redox mediators. These molecules should possess a redox potential that is close to that of the halogen redox couple, acceptable water solubility and high stability, especially under neutral or weakly acidic conditions. Among various organic molecules (Table 2), TEMPOs and their derivatives are promising candidates for optimizing the conversion pathway of high-valent halogens [109]. TEMPOs exhibit a high redox potential that aligns closely with high-valent halogen conversion and demonstrate excellent water solubility under neutral conditions [110]. Finally, increasing the mass loading and areal capacity of the cathode is crucial for enhancing the energy density of AZHBs systems. Scaling up the electrolyte of the coin cell to the thick cathode in a grid scale poses a huge challenge. Consequently, the advancement and development of high wettability and lean electrolyte systems deserve further attention.

**Table 2.** Summary of redox-active organic species reported in aqueous batteries.

Redox-active organic molecules	Supporting electrolytes	Redox potential	Solubility	Ref.
TEMPO/Phenazine	NaCl	0.6 V vs. Ag/AgCl	NA	[111]
Viologen/TEMPO	NaCl + buffer	0.67 V vs. Ag/AgCl	1 g L^−1^	[112]
Fe(CN)_6_^4−^	ZnSO_4_ + PVA	0.6 V vs. SHE	NA	[113]
(2,7-AQDS)/NaI	NaI + I_2_ + Na_2_SO_4_ catholyte	0.62 V vs. SHE	0.1 M	[114]
K_4_Fe(CN)_6_^4−^	KCl catholyte	0.45 V vs. SHE	0.8 M	[115]
N^Me^–TEMPO	NaCl	0.94 V vs. NHE	0.5 M	[116]
Pyr–TEMPO	ZnCl_2_ + NH_4_Cl	0.84 V vs. SHE	0.1 M	[117]
TEMPTMA	NaCl	0.79 V vs. Ag/AgCl	3.2 M	[118]
FcNCl	NaCl	0.61 V vs. NHE	4 M	[119]
(SPr)_2_V	KCl	–0.43 V vs. NHE	2 M	[120]
DHPS	KOH	–1 V vs. Ag/AgCl	0.1 M	[121]
DHBQ	KOH	–0.7 V vs. SHE	4.3 M	[122]
FQH_2_/FQ	H_2_SO_4_	0.7 V vs. SHE	1.4 M	[123]
THAQ	KOH	− 0.86 V vs. Ag/AgCl	1.88 M	[124]

## PROPOSED ROUTES FOR SYNCHRONOUS ELECTROLYTES

Given the above-mentioned design principles of electrolyte structures and components for synchronous electrolytes, we suggest three potential electrolyte systems to achieve further synchronous optimization in AZHBs: biphasic electrolytes, gradient hydrogel electrolytes and ionic liquid electrolytes.

### Biphasic electrolytes

In a biphasic electrolyte, the electrolyte will spontaneously form two phases under thermodynamic drive and maintain a clear interface even under external force disturbance. During the process of charging and discharging, the corresponding soluble active substances of the anode and the cathode will be enriched in respective phases and the charge balance can be maintained through the reciprocating migration of mediator ions [91]. This unique decoupling advantage enables the construction of a synchronous electrolyte. The formation of this system originates from active and passive phase separation. Active phase separation is initiated by disparities in the polarity and density between two types of immiscible solvents, which can be described and selected by using Hildebrand's solubility parameter [125] whereas passive phase separation relies on the salt-out effect. Initially, the multi-solvent system is miscible and homogeneous. The strong solvation effect between the added anions/cations and the high-polarity solvents leads to the displacement of the low-polarity solvent, ultimately resulting in the occurrence of phase separation [126].

**Figure 5.**
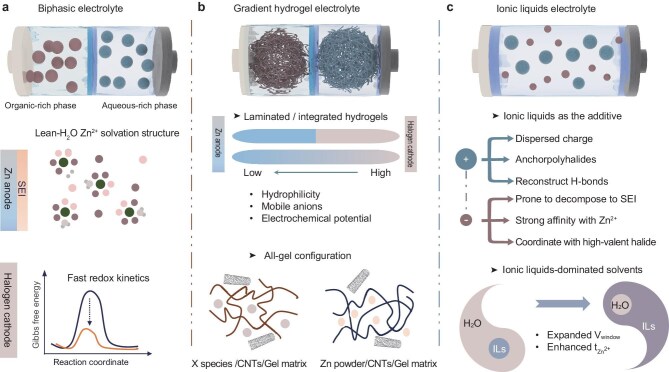
Potential candidates of synchronous electrolytes for AZHBs. (a) Biphasic electrolytes, (b) gradient hydrogel electrolytes and (c) ionic liquid electrolytes.

An optimal AZHB could employ biphasic electrolytes that comprise an organic-dominated phase and a water-dominated phase (Fig. 5a). The presence of the organic-dominated phase can suppress the water-related side reactions on the anode side and facilitate the formation of the SEI, leading to coulombic efficiencies of >99.9%, as verified in organic systems such as polycarbonate [127] and acetonitrile [128]. The water-dominated phase on the cathode side not only allows fast Zn^2+^ redox kinetics, but also confines the halogen active substances and suppresses the shuttling of the polyhalides. Despite the merits of phase-separated biphasic electrolytes, it is still challenging to discover an appropriate combination for the organic solvents–zinc salt. Common zinc salts show remarkably low levels of solubility in alkanes, carbonates and ether electrolytes. Soluble organic anions such as TFSI^−^ are not preferable because of high cost. This problem could be addressed by replacing them with appropriate hydrotropic agents to increase the solubility of the zinc salts, which has been demonstrated in acetate-based [129], amide-based [130] and perchlorate-based electrolytes [42]. In addition, certain hydrophobic ionic liquids can serve as solvents for the organic phase, providing hydrophobicity while facilitating the transport of Zn^2+^. Such biphasic architecture has been attempted in zinc–halogen batteries [131]. For biphasic electrolytes based on passive phase separation, ethanol, NMP, tetrahydrofuran, DMF and DMSO can be considered as candidate solvents for the organic phase. Numerous studies have been conducted to demonstrate the benefits of these co-solvent components in regulating the Zn^2+^ solvation structure and constructing the SEI [132–137]. As for the choice of zinc salts, according to the Hofmeister series [138], anions such as SO_4_^2−^, PO_4_^2−^, OAc^−^, F^−^, Cl^−^ and Br^−^, with small sizes and concentrated charge distribution, are suitable as salt-in components for the water phase, while anions such as Bph_4_^−^, CF_3_SO_3_^−^, SCN^−^, BF_4_^−^, ClO_4_^−^, PF_6_^−^, I^−^ and TFSI^−^ might be considered potential candidates for the organic phase due to their large and polarizable nature. These characteristics make them easily separatable from the water phase.

### Gradient hydrogel electrolyte

The main advantage of hydrogels lies in the designable functional groups and flexible combinations. In AZHBs, properties gradients including mobile charge, Zn^2+^ transference number, hydrophilicity, electrochemical potential, ionic conductivity, etc. can be constructed. This design aims to fulfill the precise demands of the zinc metal and halogen electrodes, which can be achieved with a multilayer stack of homogeneous hydrogels, i.e. laminated hydrogels or single layers of hydrogels with functional gradients, i.e. integrated hydrogels (Fig. 5b). Laminated hydrogels are easy to manufacture and highly flexible in design, allowing the combination of different hydrogel properties, but they suffer from interface instability and additional interfacial resistance. In contrast, integrated hydrogels with functional gradients offer consistent properties and smooth ion migration. However, it is challenging to achieve uniformity and repeatability in the gradient distribution.

For the Zn-anode side, hydrogel systems with low water content are preferred, allowing an expanded electrochemical window and a notable inhibition in water-related side reactions and HER [139]. On the halogen side, high ionic conductivity is essential to maintain fast reaction kinetics. In addition, anions in the hydrogel that are close to the halogen side are also critical to activate and stabilize the conversion of high-valent halides. Accordingly, a loose polymer network with a large number of water molecules, high hydrophilicity, rich anions and a moderate Zn^2+^ transference number is favorable for the halogen cathode. Such gradient structures can be achieved by merging two heterogeneous hydrogels or manipulating the distribution of hydrophilic and hydrophobic monomers in the precursor solution [140,141]. Another vital gradient parameter is the mobile charge. For the Zn-anode side, the polyanionic network with a high Zn^2+^ transference number can efficiently eliminate the side reactions that are involved with anions. Moreover, the fixed negative charges on the network can serve as zincophilic sites to homogenize the Zn^2+^ flux and guide uniform deposition [142]. In contrast, polycationic hydrogels or neutral hydrogels with halogen-philic sites are suitable for the halogen side. Nevertheless, they must possess an ultra-thin structure or be seamlessly incorporated within the cathode to guarantee the reutilization of the active materials. Similarly, this mobile charge gradient can be achieved by using laminated hydrogels with different charges. Additionally, the gradient of mobile charge can be established with the aid of diffusion or an external field [143,144]. The electrostatic field or chemical potential can be applied to the pre-hydrogel solution during the polymerization to achieve the asymmetric distribution of anions and cations, thereby fabricating the integrated hydrogel with a mobile charge gradient. The electrochemical potential is another gradient parameter that is worth taking into account. Combining hydrogels with high anode stability and high cathode stability can further broaden the electrochemical window. This concept can be exemplified in lithium-ion batteries in which ionogels comprising ionic liquids with distinct redox potentials are employed [145]. Furthermore, hydrogel electrolytes feature an inherent benefit in the application of flexible devices. All-gel zinc–halogen batteries can be constructed by mixing electrode active materials, conductive fillers and gel precursor solutions. Unique properties such as self-healing, stretchability and implantability can be introduced into AZHBs through monomer engineering [146].

### Ionic liquid electrolyte

The final candidate for a synchronous electrolyte is the ionic liquid electrolyte (Fig. 5c), which refers to the aqueous electrolyte containing ionic liquid additives or ionic liquids as the dominant solvent. Ionic liquids are organic salts that are composed of organic cations and inorganic/organic anions, which possess unique properties such as high electrochemical stability, low flammability and high fluidity [147]. The size asymmetry endows them with weakly associated characteristics, implying that cations and anions in ionic liquids can independently participate in solvation, absorption, diffusion and conversion processes. This inherent decoupling advantage of a single component allows the targeted optimization to be realized in AZHBs through customizable anions and cations. For Zn anodes, abundant hydrogen-bonding sites in ionic liquids enable the reconstruction of hydrogen-bonding networks [148]. More importantly, the anions of ionic liquids can be utilized for SEI construction and solvation structure modulation [149], while the charge-dispersed cations play a critical role in IHP regulation [150]. For halogen cathodes, polyiodide ions can combine with imidazolium cations and specific quaternary ammonium ions to form water-insoluble and high-conductive ionic liquids [105]. This new type of ionic liquid suppresses the shuttle effect and dissolution of iodine cathodes while maintaining rigorous electrochemical activity. This polyiodide ionic liquid exhibits high ionic and electronic conductivity, effectively functioning as an auxiliary current collector. It readily accepts ions from the electrolyte and electrons from the electrode, resulting in low transfer resistance and accelerated reaction kinetics. It is conceivable to extend ionic liquids to Zn–Br_2_ or Zn–Cl_2_ systems as additives or solvents to alleviate the migration and formation of halogen intermediates. However, excessively high concentrations of the polyhalide ionic liquid can reduce the ionic conductivity of the electrolyte and deteriorate the deposition behavior on the Zn side. Furthermore, multi-electron conversion can be initiated and stabilized by using halogen-based ionic liquids, such as chloride-ionic liquids or bromide-ionic liquids. These halogen ions can engage in the solvation structure to suppress water decomposition and firmly anchor high-valent halides to avoid hydrolysis. Finally, ionic liquids that contain electron-donating groups, such as O^−^ and –NR₂, can also be considered to activate the halogen conversion towards a high-valent state. Hence, ionic liquids containing [H_2_NCOO]^−^, [H_2_NSO_3_]^−^, [H_2_NCH_2_COO]^−^ and [H_2_NPO_4_]^−^ are expected to form stable complexes with positive halogen ions.

Another system of interest is the high-concentration electrolyte system, which increases the transference number of metal ions and diminishes water reactivity [129]. As ionic solvents, ionic liquids can act as solvents for zinc salts. Moreover, ionic liquids, such as EMIMOAc, EMIMOTf and EMIMTFSI, have been reported to function as hydrotropic solubilization agents, increasing the maximum solubility of lithium salts in water-in-salt systems [151,152]. In this context, we suggest that concentrated electrolytes that contain zinc salts and ionic liquids are worthy of further exploration, as one may achieve higher-concentration electrolytes with a wider electrochemical stability window.

## CONCLUSION AND OUTLOOK

In the past few years, tremendous advancements have been made in AZHBs. The intrinsic thermodynamic shortfalls of Zn metal and halogens are the major reasons for their limited reversibility and longevity. Instead of depositing protection layers, which will introduce additional steps in the battery fabrication, efforts should probably be put into electrolyte optimization. In the future research of AZHBs, we call for attention on the synchronous electrolyte design to mitigate issues on both the Zn-metal anode and the halogen cathode. Our overall conclusion and suggestions are summarized below:

- Regarding the Zn anode, the plating/striping reversibility is affected by multiple factors, including the cation solvation structure, SEI, interfacial adsorption and water activity (hydrogen-bond network). These factors determine the corrosion kinetics and deposition kinetics. Accordingly, an optimized electrolyte should contain various active components, each functioning independently or synergistically. We think that the former is practically more feasible than the latter. For this, the so-called cocktail strategy [153] and high-entropy electrolytes [154] could provide meaningful solutions. Additionally, attention should be given to the reversibility of the Zn metal under rigorous testing conditions. With an expanded Zn-anode size and reduced thickness of the foil, strategies for realizing long-cycle-life Zn symmetrical cells in many reports may become invalid. Consequently, it is crucial to present CE, calendar life and reversibility as key descriptors, especially under the condition of large sizes and high Zn utilizations.
- For the halogen cathode side, it is necessary to intensify the halogen reaction kinetics, as they govern mass and electron transfer at the cathode interface. Accelerated reaction kinetics will benefit the conversion efficiency and utilization of active halides, which is important in electrodes with high mass loadings. Ideally, halogen-conversion reactions should be entirely confined within the electron-rich interface. Hence, a composite interface that consists of halide-confined electrolytes and conductive electrodes is preferable. In addition to restricting polyhalide migration through electrostatic or adsorption effects, constructing strong catalytic centers at the interface is also an efficient strategy for reducing the energy barrier of conversion reactions. For example, hydrogels with polarized groups have been attempted as metal-free OER catalysts [155], which could be employed to boost the cathode reaction kinetics.
- A further increase in the capacity and energy density calls for the multi-electron conversion of halogens. Overall, the underlying redox mechanisms for multi-electron conversion reactions are still ambiguous. For example, multi-electron reactions in iodine systems are commonly activated by chloride salts, in which it has been believed that chlorine acts merely as a coordinating ion but does not contribute additional capacity. This viewpoint needs further verification. It is also unclear whether this approach is particularly suitable for iodine-based systems. The concept of using highly electronegative ligands to activate multi-electron reactions is worth considering. Interhalogen compounds, halide and amide groups may activate multi-electron reactions in Br-based and Cl-based AZHBs by employing the sites or functional groups with high electronegativity, particularly in dilute electrolytes.
- To streamline the development of synchronous electrolytes, advanced simulations and deep *in situ* characterizations are helpful. The selection of zinc salts, co-solvents, anchoring agents and extra additives in synchronous electrolytes can be screened by using descriptors such as binding energy, solvation energy, adsorption energy, LUMO energy levels, etc. For this purpose, trial-and-error should not be continued as the major research method. Fast screening by data-driven prediction equipped with machine learning is becoming the holy grail. Moreover, combining spatial and temporal-scale characterization with spectral analysis will provide a profound comprehension of the solvation evolution and/or real-time tracking of the halogen conversion. For example, ultrafast quasi-elastic neutron scattering enables the acquisition of solvation dynamics at nanosecond timescales [156]. Additionally, the overall high performance of the AZHB will also benefit from concurrent optimization of other parts such as the host, separator and solid–liquid interfaces.

We hope that this review offers useful insights into further improvement and large-scale application of AZHBs with extended lifespan and energy density. We also have faith in stimulating the exploration of additional metal anodes (e.g. Mn, Al and Sn) coupled with conversion-type cathodes (e.g. S, Se and Te) to expand the diversity of aqueous batteries for static energy storage.
